# Unseen crisis: the unexpected face of acute haemorrhagic pancreatitis

**DOI:** 10.1093/jscr/rjaf804

**Published:** 2025-10-07

**Authors:** Anika Nathaniel, Andrew C Ekwesianya, Wing Y Chan, Josephine Mollier, Tarek Mehaina, Abraham Jesudoss, Abaraham A Ayantunde

**Affiliations:** Department of General and Colorectal Surgery, Southend University Hospital, Prittlewell Chase, Westcliff-on-Sea SS0 0RY, United Kingdom; Department of General and Colorectal Surgery, Southend University Hospital, Prittlewell Chase, Westcliff-on-Sea SS0 0RY, United Kingdom; Department of General and Colorectal Surgery, Southend University Hospital, Prittlewell Chase, Westcliff-on-Sea SS0 0RY, United Kingdom; Department of General and Colorectal Surgery, Southend University Hospital, Prittlewell Chase, Westcliff-on-Sea SS0 0RY, United Kingdom; Department of General and Colorectal Surgery, Southend University Hospital, Prittlewell Chase, Westcliff-on-Sea SS0 0RY, United Kingdom; Department of General and Colorectal Surgery, Southend University Hospital, Prittlewell Chase, Westcliff-on-Sea SS0 0RY, United Kingdom; Department of General and Colorectal Surgery, Southend University Hospital, Prittlewell Chase, Westcliff-on-Sea SS0 0RY, United Kingdom

**Keywords:** acute pancreatitis, haemorrhagic ascites, pseudoaneurysm, pancreatic necrosis, pseudocyst

## Abstract

Acute necrotising pancreatitis can lead to severe vascular complications, including venous thrombosis, pseudoaneurysm, and haemorrhage. Acute haemorrhagic pancreatitis, though rare, is life-threatening. Contrast-enhanced computed tomography (CT) scan is the preferred diagnostic tool, with image-guided embolisation as the primary treatment for bleeding vessels. Surgery may be necessary when radiological methods fail. A 39-year-old male with recurrent abdominal pain, distension, and weight loss was initially misdiagnosed with intra-abdominal malignancy based on CT findings of omental deposits. Elevated amylase levels and haemorrhagic ascetic fluid prompted further investigations. A rapid haemoglobin drop and clinical deterioration led to diagnostic laparoscopy, confirming acute haemorrhagic pancreatitis. This case highlights the diagnostic challenges of this condition, which may present subtly and evade standard imaging, resulting in delayed treatment. Clinicians should suspect haemorrhagic pancreatitis in patients with acute abdominal pain, elevated amylase or lipase, and ascites. Early recognition and intervention are crucial for better outcomes.

## Introduction

Acute pancreatitis is an acute inflammatory process of the pancreas that usually involves the surrounding tissues and sometimes remote organs. Diagnosis is based on the modified Atlanta criteria, which require at least two of three parameters: abdominal pain typical of pancreatitis, serum amylase or lipase levels elevated at least three times above normal, and imaging evidence supporting acute pancreatitis [1].

According to a 2021 UK national cohort study, gallstones, alcohol and idiopathic factors account for 50.6%, 17.5%, and 22.4% of the aetiology of acute pancreatitis, respectively. Using the modified Atlanta severity scoring criteria, severe pancreatitis occurs in 5.7% of the patients, with a 30-day mortality rate of 2.3% [2].

Vascular complications of acute pancreatitis may arise from venous thrombosis affecting the portal, splenic, or superior mesenteric veins, or from arterial involvement leading to significant haemorrhage. Acute haemorrhagic pancreatitis, a distinct subtype of severe acute pancreatitis, may result from pseudoaneurysm rupture, haemorrhage into a pseudocyst, or capillary and small vessel bleeding, with mortality up to 34%–52% [3]. Acute haemorrhagic pancreatitis is a recognized cause of sudden death [4].

This study aims to present a case of acute severe haemorrhagic pancreatitis that eluded accurate diagnosis and timely treatment despite initial biochemical and radiological evaluations. It also underscores the critical need for maintaining a high index of suspicion for this condition and emphasizes the importance of early intervention to manage bleeding and mitigate mortality risk.

## Case report

### Clinical history and examination

A 39-year-old gentleman presented to the emergency department (ED) under the medical team with a 4-week history of progressive abdominal distension, pain and weight loss. He had recently been discharged from ED a month earlier with a similar presentation, after being referred by his GP for suspected sub-acute bowel obstruction and later discharged after no evidence was found on abdominal X-ray. He denied any melaena, vomiting or constipation and had an insignificant alcohol history. He had no other significant medical history or previous surgeries.

The abdomen was firm and distended, with generalized tenderness. There was pitting pedal oedema bilaterally, but observations were stable with the vital signs within the reference range. Rectal examination was unremarkable, except for haemorrhoids.

### Investigations and treatment

Laboratory bloods showed normal liver and renal function tests, C-reactive protein 124 mg/L (normal <5), White cell count 10.4 × 10^12^/L (range: 4.0–11.0), haemoglobin 127 g/L (range: 130–180), calcium 2.4 mmol/L (range: 2.2–2.6), and a raised amylase 1039 U/L (normal <100 U/L).

**Figure 1:**
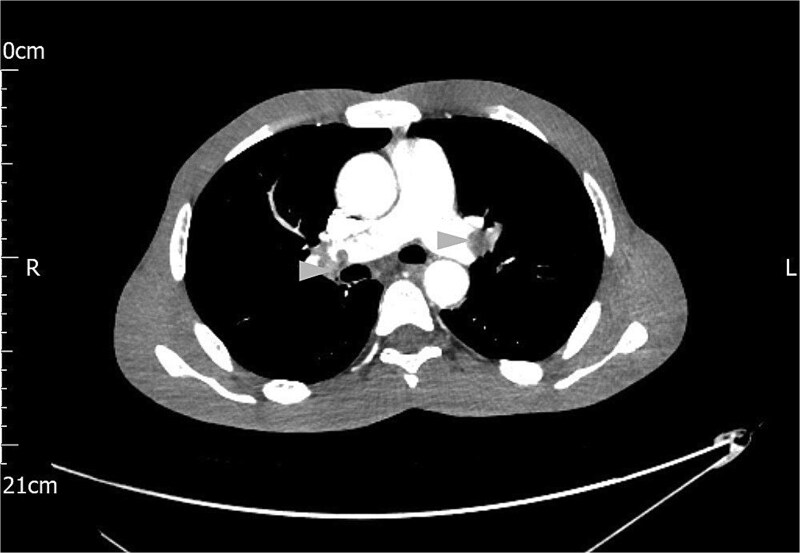
Contrast CT scan of the chest showing bilateral pulmonary artery thromboemboli (arrow heads).

A computed tomography (CT) scan of the chest, abdomen and pelvis reported gross ascites, irregularity of the tail of pancreas not typical of pancreatitis, mesenteric soft tissue density, and bilateral thromboemboli in the main pulmonary arteries, as shown in Fig. 1.

An ascetic drain was performed by the gastroenterology team, draining about 6 L of blood-stained fluid. A diagnosis of malignant effusion was entertained owing to mesenteric density changes on the CT which were suggestive of metastatic disease; however, cytology from this fluid showed no malignant cells.

**Figure 2:**
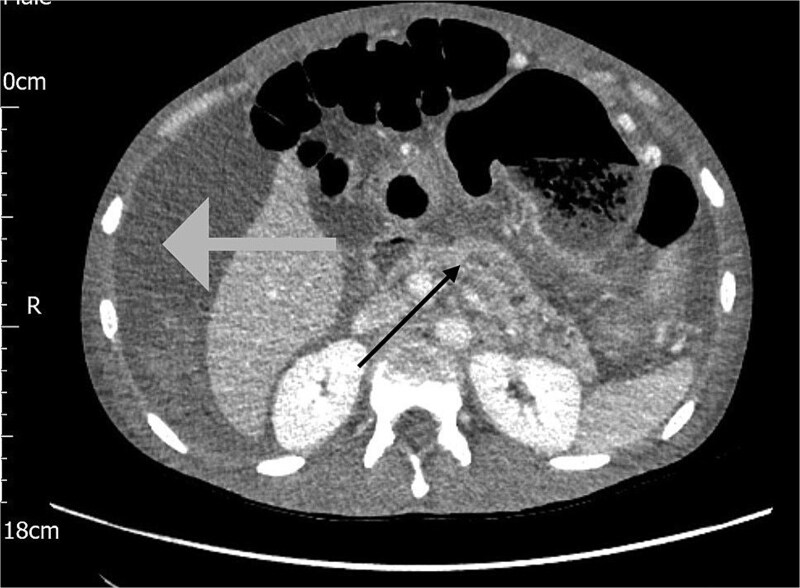
CT scan of the abdomen showing haemorrhagic ascites in the perihepatic area (grey arrow) and an irregular dilated pancreatic duct (black arrow).

The following day, the ascites recurred, accompanied by a significant drop in haemoglobin from 127 to 81 g/L. A repeat CT Abdomen Pelvis was performed which revealed a large volume of haemorrhagic ascites with no active extravasation, and an irregular dilated pancreatic duct, as shown in Fig. 2.

Abdominal paracentesis confirmed haemorrhagic fluid. Due to diagnostic uncertainty and concerns of possible vascular injury during the previous ascetic drainage procedure, the on-call surgical team was invited.

**Figure 3:**
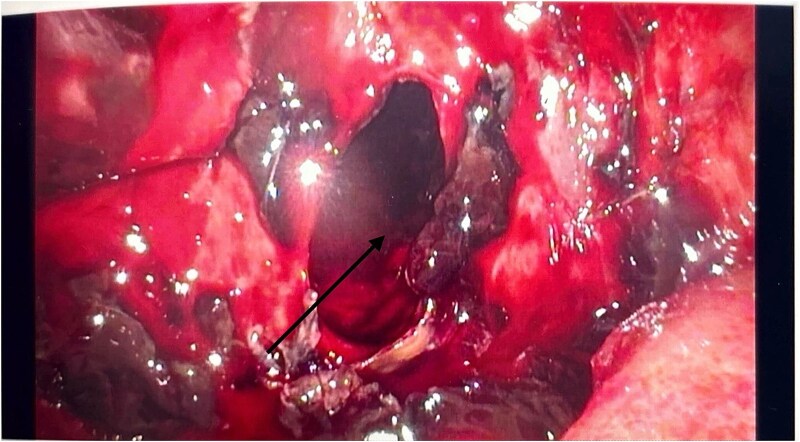
Intraoperative laparoscopic picture showing a haemorrhagic cavity in the retroperitoneum extending into the pancreas (black arrow).

He had an emergency diagnostic laparoscopy. The intraoperative findings included four quadrant haemoperitoneum, ileus involving the stomach, small and large bowels, pelvic floor peritoneal nodules, and a cavity in the retro-colic posterior peritoneum over the pancreas extending into the pancreatic parenchyma with oozing of blood, as shown in Fig. 3.

**Figure 4:**
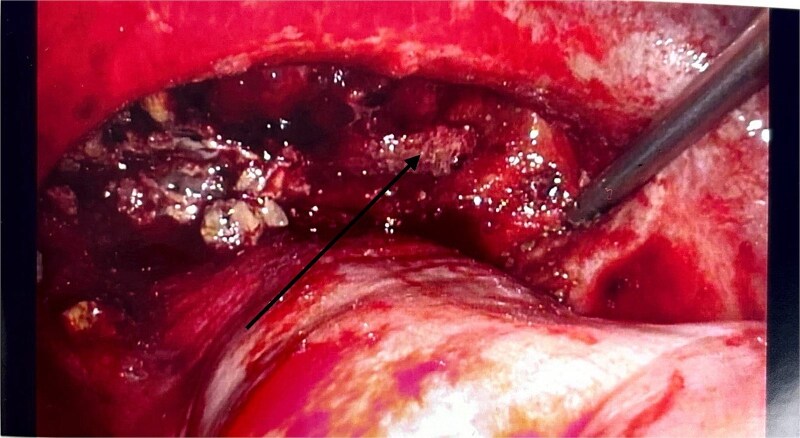
Intraoperative laparoscopic image showing the cavity packed with Surgicel haemostatic gauze (black arrow).

There were no other obvious signs of peritoneal or omental disease. About 2.6 L of haemoperitoneum with clots was drained during the surgery and the oozing cavity was packed with Surgicel haemostatic gauze, as shown in Fig. 4.

Postoperatively, the patient was transferred to the regional hepatopancreatobiliary centre at Royal London Hospital, where he was admitted in the critical care unit and recovered without further surgical intervention. He was later discharged on long term direct oral acting anticoagulation. The patient was discussed in the Upper Gastrointestinal Multidisciplinary Team meeting, where follow-up CT scan, magnetic resonance cholangiopancreatography (MRCP) and endoscopic ultrasound (EUS) were recommended. The EUS reported heterogeneity of the pancreatic tissue and the pancreatic duct with signs of chronicity and calcification, while the MRCP showed resolution of the pancreatic duct dilatation. He was later discharged on the patient initiated follow-up pathway, with no further intended surveillance scans or procedures.

## Discussion

Arterial complications occur in 1%–10% of patients with acute pancreatitis. The acute inflammatory response and pancreatic necrosis, amplified by cytokines produced during the inflammatory process, result in leakage of enzyme-rich pancreatic juice. These proteolytic and lipolytic enzymes erode blood vessel walls, potentially causing pseudoaneurysm formation or direct vessel wall disruption. Significant haemorrhage may result from pseudoaneurysm rupture, enzymatic degradation of arterial walls, or rupture of a pseudocyst wall [3].

In pancreatitis, bleeding may manifest as haemoperitoneum (into the peritoneal cavity), haemosuccus pancreaticus (into the bowel lumen via the pancreatic duct), or within a pseudocyst. The intense pain from necrotising pancreatitis can mask symptoms of haemoperitoneum, making diagnosis challenging. Cullen’s and Grey Turner’s signs, indicative of retroperitoneal bleeding, typically appear only in the advanced stages of the haemorrhagic process. The presence of haemorrhage in acute pancreatitis is independently associated with a worse prognosis [5]. Therefore, heightened clinical suspicion, routine haemoglobin monitoring, and contrast-enhanced imaging are critical to detect this life-threatening condition.

Our index patient highlights the challenges of accurately diagnosing acute haemorrhagic pancreatitis. Despite a significantly elevated amylase level at presentation, the month-long symptom duration, significant ascites, weight loss, mesenteric density noted on CT and absence of a definitive pancreatitis diagnosis on the initial CT report, shifted the focus away from pancreatitis in favour of an intra-abdominal malignancy. Consequently, the patient was under the care of physicians rather than the surgeons who typically manage acute pancreatitis patients in the hospital.

Vascular complications of acute pancreatitis are usually detected by triple-phase contrast CT scan, although an MRI scan would more accurately delineate pancreatic necrosis and its local sequelae [6, 7]. The preferred treatment of haemorrhagic pancreatitis or a pancreatic pseudoaneurysm is transcatheter arterial embolisation via interventional radiology. Laparoscopy or laparotomy is reserved for cases in which there is failure of accurate radiological diagnosis or adequate endovascular treatment [8].

The bloody ascites, rapid recurrence within 24 hours post-drainage, a sharp drop in haemoglobin and a repeat CT scan reporting haemoperitoneum also did not heighten the suspicion of acute haemorrhagic pancreatitis. Instead, a surgical referral was prompted by concerns that the prior ascetic drainage procedure might have injured a major vessel. The definitive diagnosis of pancreatitis was only established on laparoscopy, during which the bleeding was also controlled.

## Conclusion

Acute haemorrhagic pancreatitis is an uncommon but potentially life-threatening complication of acute pancreatitis. Treatment heavily relies on diagnostic imaging to identify sources of bleeding, allowing for less invasive interventional radiological approaches to management of these critically ill patients. However, as demonstrated in the case study, it is not always identified through imaging as no active extravasation was identified on the CT report of our patient, and hence a diagnostic laparoscopy was required to ultimately save this patient’s life.
